# Evaluation of CI electrode position from imaging: comparison of an automated technique with the established manual method

**DOI:** 10.1186/s12880-023-01102-6

**Published:** 2023-09-29

**Authors:** Alexander Mewes, Christopher Bennett, Jan Dambon, Goetz Brademann, Matthias Hey

**Affiliations:** 1 Department of Otorhinolaryngology, Head and Neck Surgery, Universitätsklinikum Schleswig-Holstein (UKSH), Campus Kiel, Arnold-Heller-Straße 3, 24105 Kiel, Germany; 2 Cochlear Ltd., Sydney, Australia

**Keywords:** Cochlear implant, IGCIP, Electrode localization, Electrode-to-modiolus-distance, EMD, Angular depth of insertion, aDOI; imaging

## Abstract

**Background:**

A manual evaluation of the CI electrode position from CT and DVT scans may be affected by diagnostic errors due to cognitive biases. The aim of this study was to compare the CI electrode localization using an automated method (image-guided cochlear implant programming, IGCIP) with the clinically established manual method.

**Methods:**

This prospective experimental study was conducted on a dataset comprising *N*=50 subjects undergoing cochlear implantation with a Nucleus® CI532 or CI632 Slim Modiolar electrode. Scalar localization, electrode-to-modiolar axis distances (EMD) and angular insertion depth (aDOI) were compared between the automated IGCIP tool and the manual method. Two raters made the manual measurements, and the interrater reliability (±1.96·SD) was determined as the reference for the method comparison. The method comparison was performed using a correlation analysis and a Bland-Altman analysis.

**Results:**

Concerning the scalar localization, all electrodes were localized both manually and automatically in the scala tympani. The interrater differences ranged between ±0.2 mm (EMD) and ±10° (aDOI). There was a bias between the automatic and manual method in measuring both localization parameters, which on the one hand was smaller than the interrater variations. On the other hand, this bias depended on the magnitude of the EMD respectively aDOI. A post-hoc analysis revealed that the deviations between the methods were likely due to a different selection of mid-modiolar axis.

**Conclusions:**

The IGCIP is a promising tool for automated processing of CT and DVT scans and has useful functionality such as being able to segment the cochlear using post-operative scans. When measuring EMD, the IGCIP tool is superior to the manual method because the smallest possible distance to the axis is determined depending on the cochlear turn, whereas the manual method selects the helicotrema as the reference point rigidly. Functionality to deal with motion artifacts and measurements of aDOI according to the consensus approach are necessary, otherwise the IGCIP is not unrestrictedly ready for clinical use.

## Background

Obtaining information about the intracochlear location of the electrode is of importance for the treatment of severe to profound hearing loss with a cochlear implant (CI). During the surgical phase, the surgeon must know whether the electrode has been placed inside of the cochlea as intended. Prior to initial stimulation, the audiologist must be aware of the number of electrodes inserted into the cochlea and whether there is the presence of a tip fold-over. A tip fold-over is efficiently visualized with an X-ray image, however, other placement characteristics of clinical benefit are not able to be derived from X-ray imaging. To detect an incomplete insertion, it is helpful to measure the angular depth of insertion (aDOI) at the most basal electrode relative to the round window. Measurements of aDOI can be useful to balance, or at least to better evaluate, pitch perception in bilateral CI fittings between the ears. In additional to the insertion depth, information about the scalar localization and the proximity of the electrodes to the spiral ganglion cells are also of clinical interest for speech processor fitting. In clinical practice, these two spatial parameters can be examined in circumstances where the patients*’* postoperative speech understanding is worse than expected [1–6], or if ECAP thresholds or C and T levels are pathologically high [7–17]. When considering the development of new CI electrode arrays, measurements of the electrode-to-modiolar axis distance EMD with other physiological characteristics of cochlea are of importance to verify whether the arrays are behaving as intended.

High-resolution imaging techniques such as Computer Tomography (CT) or Digital Volume Tomography (DVT) are required instead of an X-ray to evaluate electrode array placement as landmarks or structural features of cochlea are not visible in X-ray images. Spatial parameters such as EMD, aDOI and scalar localization are extracted from two-dimensional planes that are reformatted by multiplanar reconstruction of the 3D volume data set. In clinical practice this procedure is conducted by manual image-processing and may be affected by diagnostic errors (missing findings or misinterpretation of findings). Diagnostic errors in radiology commonly result from a combination of system-related factors and cognitive-perceptual biases that can be present in both experienced and inexperienced raters [18–23]. The use of accurate, automatic electrode localization techniques could be helpful in reducing such diagnostic errors. Braithwaite et al. (2016) [24] and Bennink et al. (2017) [25] proposed semi-automated approaches for locating electrode arrays, that require manual initialization. A fully automated approach for determining the electrode position was developed at Vanderbilt University (which is currently not yet approved for general clinical use). This tool was designed to localize the electrode array as accurately as possible to identify and deactivate electrode contacts that may cause undesired channel interaction (image-guided cochlear implant programming, IGCIP) [26]. The primary spatial parameter that IGCIP uses for programming modifications is the shortest distance between the center of each electrode and the modiolar surface along the length of the modiolus [26] which is different to electrode-to-modiolar axis distances (EMD). That is, the modiolar surface represents the interface between the spiral ganglion cells and the intracochlear cavities (ST, SV). In addition, other established spatial parameters such aDOI, EMD and the scalar localization are also determined automatically with the IGCIP tool. Although the automatic electrode localization within IGCIP was ensured by experts [27], there is no external verification of the validity of these spatial parameters.

The aim of this work was therefore to compare the spatial parameters EMD, aDOI and scalar localization between the automatic approach and the clinically established manual method. The research question is whether one can measure these spatial parameters and attain comparable results with either the automatic or manual method.

## Methods

### Aim, design and setting of the study

This comparison study determines if two methods (automatic and manual) measure spatial parameters for locating the CI electrode array in an equivalent manner. A prospective experimental study design was chosen. The study was conducted at a tertiary referral medical center with a cochlear-implant program. 

### Subjects

Fifty adult subjects were to be included in the study who underwent cochlear implantation with a Nucleus^®^ CI532 or CI632 Slim Modiolar electrode in the period of June 2019 to September 2021. The subjects also met the following inclusion criteria: void of adverse placements (buckles, tip fold-over); void of cochlear malformation; void of reinsertion of the array; available post-operative CT or DVT scan with isotropic voxel size ≤ 0.25 mm and no less than ¼ head scan. A total of 123 patients were available that met the criteria, from which, 29 post-operative DVT scans (24%) had to be discarded due to motion artifacts which would have prevented a clear determination of electrode position. Motion artifacts were detected by manual screening of all scans for double contours. From the remaining patients, fifty subjects for this study were randomly selected. A post-operative DVT was analyzed for each of the fifty subjects. The DVT unit of this study was the 3D eXam by KaVo (Biberbach, Germany). Patients were seated during the scanning. All scans were performed with an image voltage of 120 kV, a tube current of 5 mA with pulsed X-ray emission, and an exposition time of 7 seconds. Data on image resolution, as well as demographic and CI-related characteristics of the study population, are provided in Table 1.

**Table 1:** Demographic, CI-related and imaging characteristics of the subjects

Subject number	Ear implanted	Etiology of unilateral hearing loss	Implant type	Image matrix	Size of isotropic image voxel (mm^3^)	Image quality sum score (0 to 12)
1	L	Infection	CI632	640 x 640	0.25	4
2	R	Otosclerosis	CI632	800 x 800	0.2	5
3	R	Infection	CI632	800 x 800	0.2	7
4	L	Unknown	CI632	800 x 800	0.2	6
5	L	Syndromal	CI632	800 x 800	0.2	6
6	R	Unknown	CI632	800 x 800	0.2	5
7	R	Sudden hearing loss	CI632	800 x 800	0.2	7
8	R	Otosclerosis	CI632	640 x 640	0.25	7
9	L	Infection	CI632	800 x 800	0.2	5
10	L	Familial	CI632	800 x 800	0.2	5
11	R	Unknown	CI632	800 x 800	0.2	6
12	L	Unknown	CI632	800 x 800	0.2	7
13	L	Sudden hearing loss	CI632	800 x 800	0.2	8
14	R	Unknown	CI632	800 x 800	0.2	6
15	R	Meniere’s disease	CI532	800 x 800	0.2	5
16	L	Granulomatosis	CI532	640 x 640	0.25	6
17	R	Congenital	CI632	800 x 800	0.2	7
18	L	Sudden hearing loss	CI632	800 x 800	0.2	5
19	R	Sudden hearing loss	CI632	800 x 800	0.2	6
20	R	Unknown	CI632	800 x 800	0.2	7
21	R	Sudden hearing loss	CI632	640 x 640	0.25	7
22	L	Sudden hearing loss	CI632	800 x 800	0.2	5
23	R	Trauma	CI632	800 x 800	0.2	4
24	L	Sudden hearing loss	CI632	800 x 800	0.2	6
25	R	Meniere’s disease	CI632	800 x 800	0.2	5
26	R	Sudden hearing loss	CI632	640 x 640	0.25	6
27	R	Trauma	CI632	800 x 800	0.2	4
28	L	Ototoxic	CI632	800 x 800	0.2	7
29	L	Sudden hearing loss	CI632	800 x 800	0.2	6
30	L	Sudden hearing loss	CI632	800 x 800	0.2	8
31	L	Congenital	CI632	800 x 800	0.2	5
32	R	Otosclerosis	CI632	800 x 800	0.2	4
33	L	Otosclerosis	CI632	800 x 800	0.2	6
34	R	Unknown	CI632	800 x 800	0.2	5
35	R	Unknown	CI632	800 x 800	0.2	8
36	R	Congenital	CI632	800 x 800	0.2	7
37	L	Congenital	CI632	800 x 800	0.2	5
38	L	Unknown	CI632	800 x 800	0.2	7
39	R	Sudden hearing loss	CI632	640 x 640	0.25	8
40	R	Otosclerosis	CI632	800 x 800	0.2	7
41	R	Infection	CI532	640 x 640	0.25	5
42	R	Unknown	CI632	800 x 800	0.2	5
43	R	Meniere’s disease	CI632	800 x 800	0.2	4
44	R	Otosclerosis	CI632	800 x 800	0.2	6
45	R	Infection	CI632	800 x 800	0.2	6
46	L	Sudden hearing loss	CI632	800 x 800	0.2	8
47	R	Sudden hearing loss	CI632	800 x 800	0.2	8
48	R	Unknown	CI632	800 x 800	0.2	5
49	R	Sudden hearing loss	CI632	800 x 800	0.2	7
50	R	Unknown	CI632	800 x 800	0.2	6

### Manual image analysis

Conventional DICOM viewers (“RadiAnt DICOM viewer”, Medixant; “KaVO eXam Vision”, KaVo Dental GmbH) were used for the manual measurements and the verification of the scalar localization of the electrode array as well as for checking the image quality (visual assessment). Two experts with experience in evaluating the electrode placement measured each of the 50 image sets [7, 16, 23]. The manual CI localization was performed in accordance with a consensus panel on a cochlear coordinate system [28], in which the mid-modiolar axis is perpendicular to a plane through the basal turn of the cochlea, with the helicotrema as the origin of this axis (‘cochlear view’) [16, 28, 29]. 

**Fig. 1:**
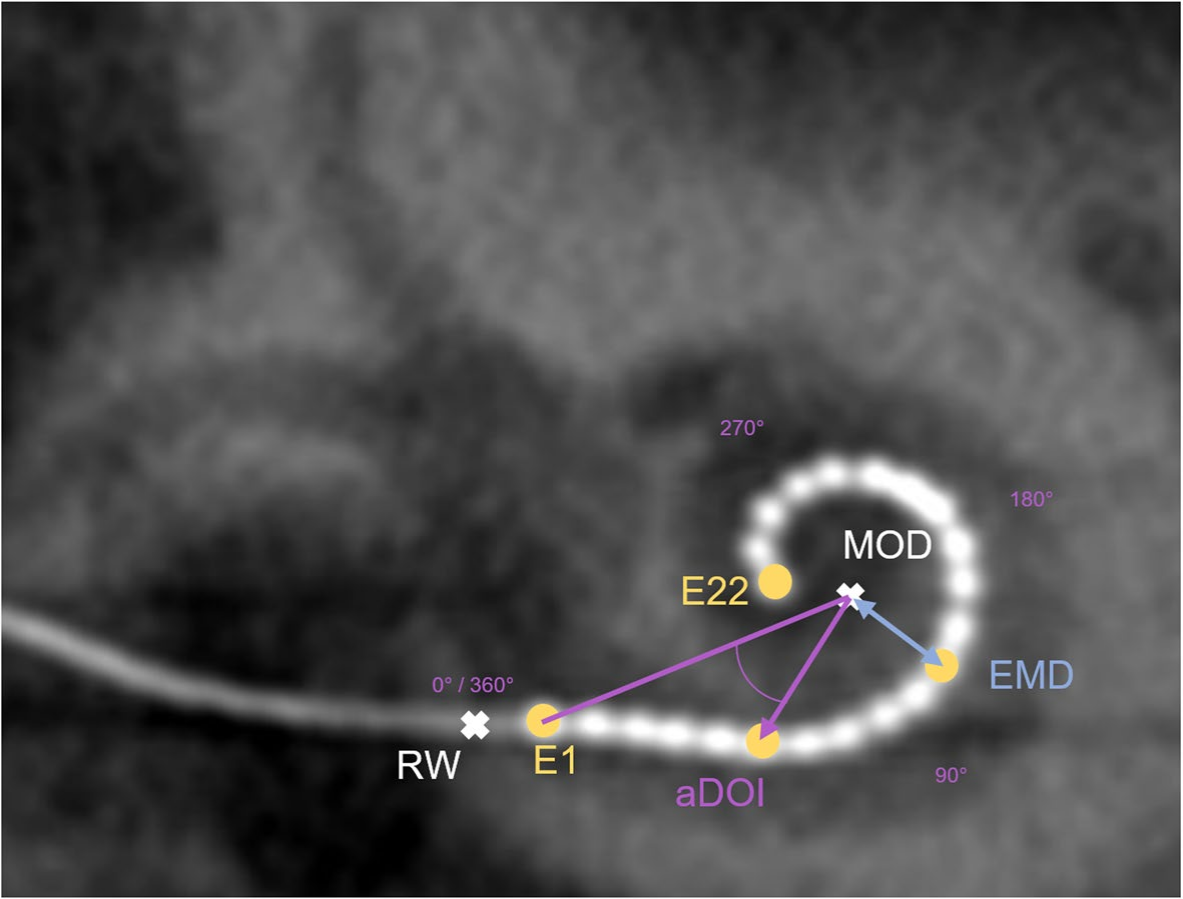
Schematic illustration of spatial parameters to be measured: aDOI, angle of insertion depth, relative to the chord produced between the mid-modiolar axis “MOD” (helicotrema) and the center of the round window “RW” (0°); EMD, distance from the center of an electrode “E” to MOD

Spatial parameters were measured and extracted from this two-dimensional cross-sectional plane, and the center of the helicotrema has been used as the mid-modiolar axis (Fig. 1). To detect the helicotrema, the 2D plane of the cochlear view was moved along the mid-modiolar axis in the direction of the apex until the helicotrema was visible. Spatial parameters included:

- aDOI_manual_, angle of insertion depth, relative to the chord produced between the mid-modiolar axis and the center of the round window (0°); the round window was directly detected visually in the cochlear view (Fig. 1).
- EMD_manual_, distance from the center of an electrode to the mid-modiolar axis.
- Scalar localization of each electrode (scala tympani or scala vestibuli).

The parameters aDOI and EMD were measured at each even-numbered electrode and at electrode E1 of the total of 22 electrodes. From our experience, EMD and aDOI measurements at every second electrode provides a good compromise between accuracy, time requirements and the clinical interest.

The interrater reliability of measuring the aDOI and EMD manually was determined for the complete study population to be able to interpret the findings of the method comparison against *a priori* criteria. That is, the two raters have measured each spatial parameter once.

The scalar localization for each electrode was determined by further processing the cochlear view: A cross-section of the intracochlear lumen (transmodiolar reformation) and a curved reconstruction of the electrode path within the cochlea (“unrolling” the cochlea) [1, 7, 30–33].

### Automatic image analysis

Automated data were calculated from DICOM measurements using the IGCIP tool. The first step in this method was to identify and to segment the anatomical structures of the inner ear (e.g., scala tympani, ST; scala vestibuli, SV; mid-modiolar axis) using post-operative DVT images [6, 34–37]. That is, a point distribution model (“atlas”) of the cochlea is nonrigidly warped to register to a new patient DVT. 

Each point of the model can be allocated to an anatomical landmark (e.g., center of the round window, RW; outer wall of the ST at 180° insertion depth). Pre-operative CT or DVT images were not available for all subjects. Post-operative DVT images were exclusively used for consistent segmentation of the cochlear anatomy [36, 37]. In a second step, the post-operative image was subsequently analyzed to locate the intracochlear electrodes [38–41] and merged with the post-operative image that included the segmented cochlear anatomy. Both the anatomy and electrode localization were visually verified to ensure that the automatic process was accurate (i.e. that all relevant anatomical structures and the electrodes had been located correctly).

By combining the two images created in steps one and two, a range of implant-to-anatomy measurements was automatically calculated and exported [42–44]. These included the following spatial parameters to be analyzed in this study:

- aDOI_auto_, angle of insertion depth of each electrode, relative to the chord produced between the mid-modiolar axis and the center of the round window (0°), see Fig. 5 in [45] as an example.
- EMD_auto_, minimal distance of the center of each electrode to the mid-modiolar axis. This is consistent with a cylindrical coordinate system where the mid-modiolar axis is the z-axis and the radius is the distance from the electrode to the axis. Fig. 1 in [41] visualizes the relationship between an CI electrode array and the modiolus exemplarily.
- Scalar localization of each electrode (scala tympani or scala vestibuli), see Fig. 5 in [45] as an example.

The EMD, aDOI and the scalar localization data were automatically calculated and exported by the IGCIP tool. 

### Confounding analysis

As a check for any confounding bias, the influence of image quality (IQ) and image resolution (matrix and voxel size) on the spatial parameters under investigation was analyzed. IQ was assessed subjectively in terms of image noise, contrast, sharpness and artifacts. For each of the landmarks round window, mid-modiolar axis, electrodes and outer wall of the cochlear, the image quality was rated on a 4-point scale: 0, nondiagnostic; 1, sufficient for diagnostic use; 2, more than basic diagnostic; 3, diagnostic without restrictions [46]. A total IQ score was calculated from the sum of the points for all four structures, ranged from 0 to 12. IQ sum score, resolution and isotropic voxel size for each DVT scan are given in Table 1. For both confounding variables (image quality, image resolution), we investigated whether there were statistically significant differences in the bias between the methods. For this, the Wilcoxon test was used to compare the central tendencies of two samples, and an ANOVA was used for comparing the central tendencies of several samples.

### Data analysis

All statistical analyses were performed using the MATLAB™ software (The MathWorks, Inc, Natick, Massachusetts). The variables analyzed were EMD, aDOI and scalar localization of each electrode, each of which measured manually and with the automatic tool. As aDOI and EMD were only measured manually at each even-numbered electrodes and E1, only these electrodes were used for the method comparison. 

The Kolmogorov-Smirnov test was applied for testing whether the data were normally distributed. The analysis of data followed a procedure in a method-comparison study as suggested by Hanneman (2008) [47]. This procedure includes examining the relationship of the corresponding paired values as well as bias and precision statistics. 

Visual inspection of scatter diagrams was conducted to examine potential relationships between the various parameters. The Pearson Product-moment correlation *r* and the confidence interval (*CoI*) were calculated for the purpose of interpretation. 

Bias and precision statistics were generated with Bland-Altman plots to determine agreement between both methods [48–50]. To obtain the bias and the precision (“limits of agreement”), the mean values and ±1.96 standard deviations were calculated to all differences between the methods. As bias and precision statistics are calculated across all data points, proportional errors do not appear in the calculation. Therefore, the percentage error was calculated to consider the proportion between the magnitude of measurements and the bias/limits of agreement quantitatively. This error was obtained by dividing the limits of agreement (upper limit minus lower limit) by the mean value of the measurements obtained with the established (manual) method [51].

The interrater reliability (±1.96 standard deviation) of both raters served as the *a priori* criterion against which the method’s bias and precision statistics were interpreted. The rater*’*s interrater reliability in measuring the spatial parameters was confirmed by both an intraclass correlation (2-way mixed-effects model, multiple raters/measurements type with absolute agreement) and a Bland–Altman analysis.

## Results

### A priori calculation

Interrater differences of each spatial parameter were calculated as the difference from two manual measurement series made by two raters. For EMD, the bias (mean value of the differences) was 0 mm and the precision (±1.96 standard deviation) was ±0.2 mm. Analyzing the aDOI revealed a bias of 0° and the precision was found to be ±10°. The interrater precision of each parameter served a priori as the maximum value that would indicate acceptable agreement between the methods and precision of the difference. The interrater reliability was conformed by an excellent [52] intraclass correlation (ICC: 0.99 and 1), and, as mentioned above, by evidence of no bias in the interrater differences.

### Method comparison of electrode location characteristics

Concerning the scalar localization, all 600 analyzed electrodes (12 electrodes × 50 arrays) were localized both manually and automatically in the scala tympani. No scalar crossing into the scala vestibuli was observed.

**Fig. 2:**
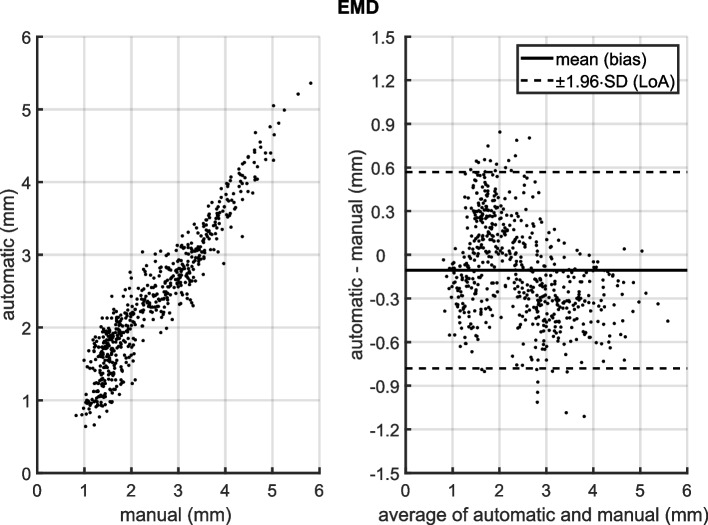
(left) Scatter diagram of electrode-to-modiolar axis distances (EMD) measured manually and with the automatic tool; (right) Bland-Altman plot of EMD with mean (bias) and ±1.96 standard deviation (limits of agreement, LoA) differences between the automatic and manual method. EMD was analyzed from *N*=50 electrode arrays at each even-numbered electrode contact from E2 to E22, as well as at E1

**Fig. 3:**
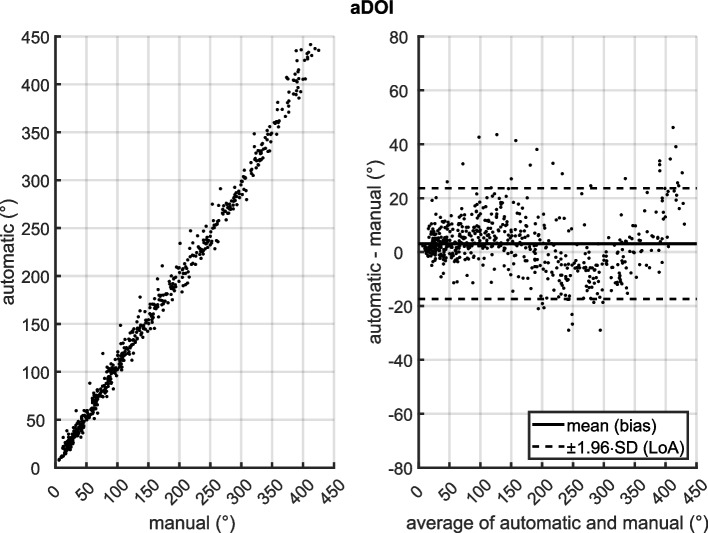
(left) Scatter diagram of insertion depth angles (aDOI) measured manually and with the automatic tool; (right) Bland-Altman plot of aDOI with mean (bias) and ±1.96 standard deviation (limits of agreement, LoA) differences between the automatic and manual method. aDOI was analyzed from *N*=50 electrode arrays at each even-numbered electrode contact from E2 to E22, as well as at E1

**Fig. 4:**
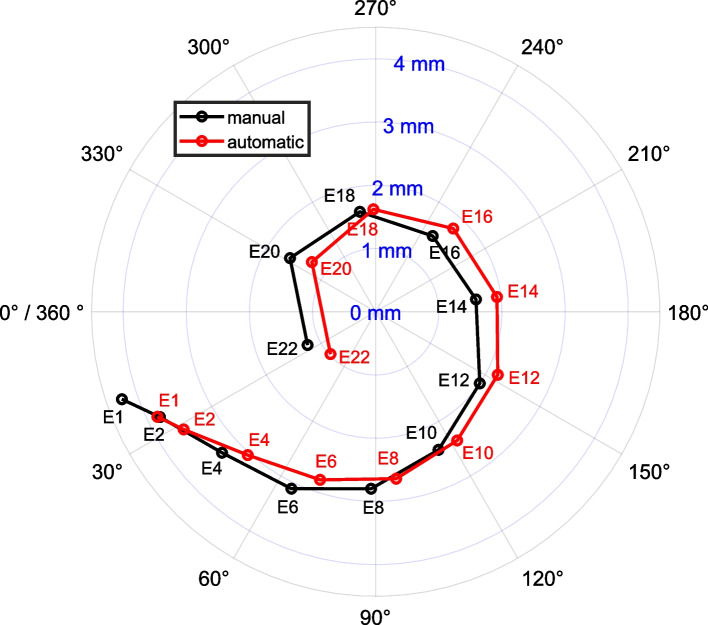
Polar plot, illustrating the mean value of EMD (radius) and aDOI (angle) with the automatic and manual method for each of the electrodes analyzed (E1, E2, E4, E6, E8, E10, E12, E14, E16, E18, E20, E22). The center of the polar plot represents the mid-modiolar axis (helicotrema) of the cochlea

Scatter diagrams and Bland-Altman plots for EMD and aDOI are shown in Figs. 2 and 3. Each Bland-Altman plot (right) represents the automatic method minus the manual method depending on the corresponding average of automatic and manual measurement, with the bias (mean of differences) and the limits of agreement (*±*1.96 standard deviation). As illustrated in Figs. 2 and 3 (left panels), the data points for both the EMD and aDOI fall near on a line of equality, suggesting there is some degree of agreement between the methods. The correlation coefficient was *r* =0.95 (95% *CoI* = [0.94, 0.95]) for EMD and *r* = 0.99 (95% *CoI* = [0.99, 1]) for aDOI, respectively, with a significance level of *p*<0.001. Regarding the Bland-Altman plots (Figs. 2 and 3, right panels), there was no clinically relevant systematic bias between the methods as the bias fall within ±1.96 standard deviation of the manual interrater differences (EMD: -0.1 mm bias compared to ±0.2 mm ±1.96·SD; aDOI: 2° bias compared to ±10° ±1.96·SD). Nonetheless, the percentage error was calculated with 55% for EMD and 26% for aDOI, indicating a clinical importance of a proportional error to the magnitude of the measurements. The difference patterns of both Bland-Altman plots, by visual observation, are partially periodic and heteroskedastic. A polar plot was created to visualize the differences in the electrode localization between both methods in an electrode-specific manner (Fig. 4). With the automatic and manual method measured, the polar plot illustrates the mean value of aDOI and EMD for each of the 12 electrodes analyzed. Visual inspection of the polar plot gives rise to a suspicion that there is a systematic bias in the electrode localization due to the selection of the mid-modiolar axis.

**Fig. 5:**
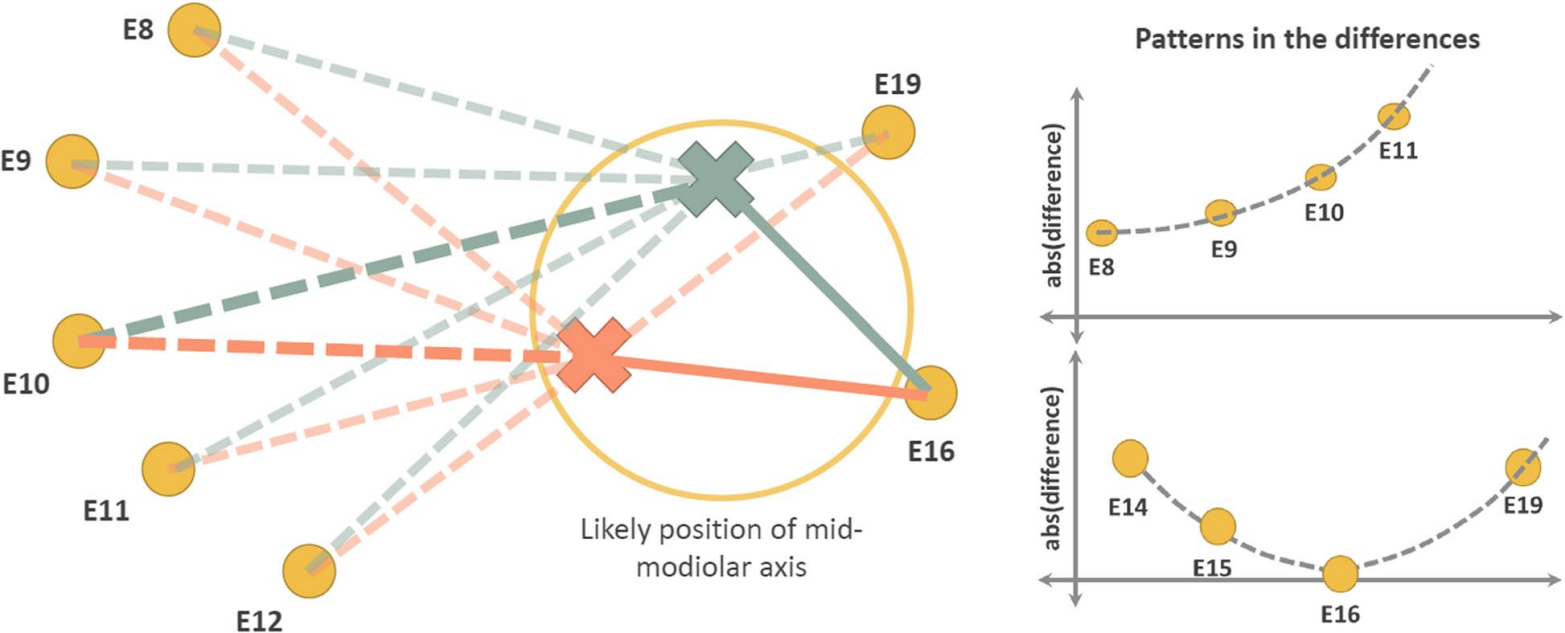
Difference pattern effect from selecting different position of the mid-modiolar axis in a hypothetical CI electrode insertion

Figure 5 presents a hypothetical scenario of an insertion of an electrode array inside of the cochlear and markups are generated using two different likely locations of the mid-modiolar axis. In this scenario the two sets of EMD are generated, one for each location of the mid-modiolar axis. Starting with electrode E8 the differences between the EMD are small. As the electrode number increases to E11, the difference between both sets of EMD increases. Thereafter, due to the way in which the electrode array conforms to the spiral shape of the cochlear duct, the differences decrease to E16 and then begin to increase. Thus, the difference in this hypothetical scenario confirm to a partial periodic pattern. In the hypothetical example displayed in Fig. 5 it was observed the variance pattern of differences between sets of EMD measurements are function of systematic differences in measurements. The systematic differences in measurements are caused by the initial section of the mid-modiolar axis of the cochlear view of the DVT scan. These systematic differences may be addressed by the adoption of uniform methods of achieving the cochlear and selecting the location of the mid-modiolar axis. Alternatively, as described in the next section, the systematic difference may be addressed by post-hoc algorithmic adjustment of electrode positions.

### Algorithm to achieve post-hoc collocation of the mid-modiolar axis

The coordinates of the electrodes were measured relative to the selected mid-modiolar axis. For repeated sets of measures where each set was measured by a different rater, the distance between the selected modioli of the different raters is unknown. Unless the selections of the mid-modiolar axis are relative to another coordinate system such as the medical image, it is not possible to directly adjust the measurements to having matching modioli. If the modioli were to be collocated, the resultant differences between the EMD of the different sets would be due to differences in the identification of the center of the round window as well as of the locations of the electrodes in the DVT scan. As described by Fig. 5, the partial periodic difference pattern is likely due to a difference in the selection of the mid-modiolar axis. The closer the modioli are collocated, the less pronounced is the partial periodic difference pattern. It can be inferred, to post-hoc achieve a better match of the modioli of set of measurements, the coordinates of a set of electrodes can be uniformly altered in such a way to minimise the magnitude of the partial period difference pattern, where the magnitude of the partial period difference pattern can be measured by Pearson’s correlation (ρ) between the sets of measurements. A correlation value closer to 0 conveys a greater difference in the selection of the mid-modiolar axis (more pronounced difference pattern), a correlation value closer (+)1 conveys a smaller difference (less pronounced difference pattern). This process can be automated by calculating the uniform alteration in coordinates by minimising a cost function via an optimisation algorithm.

1 $$EMD=\left[{EMD}_{1},{EMD}_{2},\dots ,{EMD}_{22}\right]$$

2 $$aDOI=\left[{aDOI}_{1},{aDOI}_{2},\dots ,{aDOI}_{3}\right]$$

where *EMD* is an array of EMD where each element is an individual measurement corresponds to an electrode, and *aDOI* is an array of aDOI where each element is an individual measurement corresponds to an electrode.

3 $${E}_{x}=EMD \times \mathrm{cos}(aDOI)$$

4 $${E}_{y}=EMD \times sin(aDOI)$$

where ${E}_{x}$ is an array of *x*-coordinates with 22 elements where each element corresponds to an electrode, and ${E}_{y}$ is an array *y*-coordinates with 22 elements where each element corresponds to an electrode.

5 $${E}_{x,t}={E}_{x}+{shift}_{x}$$

6 $${E}_{y,t}={E}_{y}+ {shift}_{y}$$

where ${E}_{x,t}$ is the altered *x*-coordinates, ${shift}_{x}$ is the value of the alteration on the *x*-axis, ${E}_{y,t}$ the altered *y*-coordinates, and ${shift}_{y}$ is the value of the alteration on the *y*-axis.

7 $${EMD}_{t}= \sqrt{{E}_{x,t}^{2}+{E}_{y,t}^{2}}$$

8 $${aDOI}_{t}=artan2({E}_{y,t}^{2},{E}_{x,t}^{2})$$

9 $${aDOI}_{t,i}=\left\{\begin{array}{c}{aDOI}_{t,i}+2\pi , if {aDOI}_{t,i}<0\\ {aDOI}_{t,i}\end{array}\right.,\;for\;1\le i\le 22$$

10 $${aDOI}_{t,i}=\left\{\begin{array}{c}{aDOI}_{t,i}+2\pi,if(\sum\limits_{j=1}^{i-1}{(aDOI}_{t,j}\geq\frac{3\pi}2\cap{aDOI}_{t,j}\leq2\pi\cap{aDOI}_{t,j+1}\geq0\cap{aDOI}_{t,j+1}\leq\frac\pi2))>0\\{aDOI}_{t,i}\end{array},\;for\;2\leq i\leq22\right.$$

where the ${EMD}_{t}$ is the altered *EMD*, and ${aDOI}_{t}$ is the altered *aDOI*.

11 $$\begin{aligned} cost\left({shift}_{x},{shift}_{y}\right)=\left(1-\rho ({EMD}_{t}^{se{t}_{1}},{EMD}^{se{t}_{2}})\right) \\ +\left(1- \rho ({aDOI}_{t}^{se{t}_{1}},{aDOI}^{se{t}_{2}})\right) \end{aligned}$$

where $cost\left({shift}_{x},{shift}_{y}\right)$ calculates the cost of the uniformly altered coordinates of the set 1 ($se{t}_{1}$) of the measurements.

**Fig. 6:**
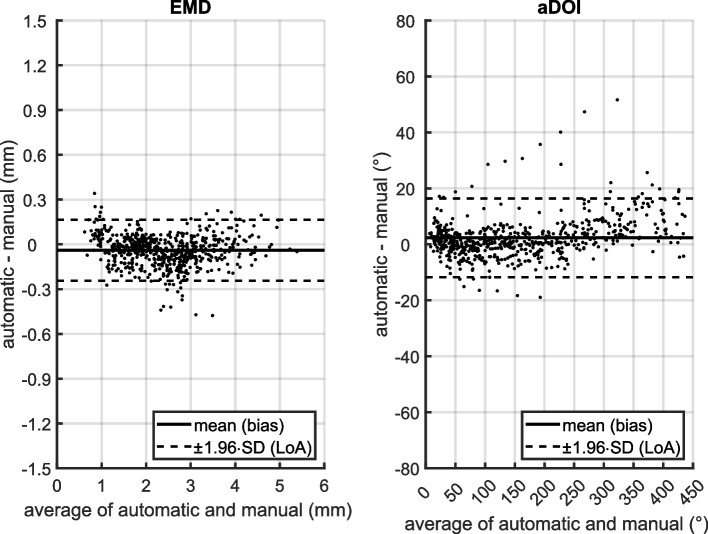
Differences between the automatic and manual method after eliminating the systematic differences may be addressed by selecting a different location of the mid-modiolar axis. Bland-Altman plots of EMD (left) and aDOI (right) with mean (bias) and ±1.96 standard deviation (limits of agreement, LoA) differences between the automatic and manual method. EMD and aDOI were analyzed from *N*=50 electrode arrays at each even-numbered electrode contact from E2 to E22, as well as at E1

Figure 6 display the Bland-Altman plot for EMD and aDOI from Figs. 2 and 3 after the optimization algorithm has been applied. For both variables, EMD and aDOI, smaller limits of agreement were obtained after optimization, i.e., a more precise agreement between both methods. The ±1.96-fold standard deviation was reduced after optimization by 71% for the EMD (0.7 mm to 0.2 mm) and by 33% for the aDOI (21° to 14°). The remaining standard deviation was thus within the range of ±1.96-fold SD for the interrater deviation of the manual method. It is then not of clinical relevance that a slight periodic pattern of aDOI differences remained even with the post-hoc algorithmic adjustment (Fig. 6, right panel).

### Confounding analysis

It was analyzed whether the confounding variables image quality score (IQ) and image resolution (IR) had an impact on the bias between both methods for each of the two spatial parameters (EMD, aDOI). The Wilcoxon test was used to compare the central bias tendencies of both IR samples (0.2 mm versus 0.25 mm voxel size) and a one-way ANOVA was used for comparing the central tendencies of the five IQ samples (IQ scores 4, 5, 6, 7 and 8). Only these five IQ scores were analyzed as the measured scores covered this range of values (frequency relative to *N*=50 subjects: IQ4 10%, IQ5 28%, IQ6 26%, IQ7 24%, IQ8 12%).

When considering the image resolution IR, there were statistically significant differences in the bias between 0.2 mm and 0.25 mm (*p*<0.001 and *p*<0.05) for both EMD and aDOI, but these differences were within the range of ±1.96-fold SD for the interrater deviation of the manual method.

In the analysis of IQ, no significant differences were found between the five IQ samples for both EMD (*p*=0.22) and aDOI (*p*=0.59) using ANOVA. Thus, neither IR nor IQ had a relevant influence on the method comparison in this study and were therefore not addressed in the further analysis.

## Discussion

CT and DVT imaging provide CI electrode array positioning and cochlear anatomical information beyond what is possible by X-ray. Current standard practice to derive this information involves manual measurements. Vanderbilt University*’*s IGCIP tool is a quasi-fully automated approach to provide information about the intracochlear electrode location to the audiologist, who is generally inexperienced in imaging and its analysis. This automated approach reduces financial and human resources by allowing audiologists to perform the evaluation with the tool themselves, rather than having it performed by a radiologist. Furthermore, automation has the potential to improve the quality of CI electrode localization by eliminating human error and improving consistency and accuracy. For general clinical use automated techniques in processing CT or DVT images are required to be as accurate and precise as manual measurements of experience experts. As far as the authors of this paper is aware the IGCIP tool is yet to be approved for use in clinical practice.

It was therefore the aim of this study to contribute to assessment of the validity of IGCIP. For this objective, spatial parameters of clinical and research interest (EMD, aDOI, scalar localization) were compared between the IGCIP approach and the clinically established approach of manual measurements.

### Methodical limitations

This study cannot assess the “accuracy” of the automated approach, because the manual approach is not a gold standard that is calibrated to be highly accurate and consistent. This work was rather a method-comparison study, comparing a less-established method with a clinically established method, and thus calculating the "bias" between the two approaches. Regardless of this limitation, the claim of the present study was to compare the automatic approach with a manual method that was as accurate as possible. For this reason, two raters with experience in evaluating CI electrode location made the manual measurements. The interrater reliability was confirmed by an excellent intraclass correlation and the ±1.96-fold SD of the EMD interrater differences was in the range of the image resolution (0.2 to 0.25 mm). It must be noted that this interrater reliability was achieved after repeated series of manual measurements, since at the study’s beginning there were some methodological differences between the raters in the detection of relevant landmarks (round window, mid-modiolar axis). To calibrate the manual method as accurate as possible, both raters had to follow the consensus panel on a cochlear coordinate system [28] exactly. It can be assumed that there are probably larger variations between different raters in the manual evaluation of the CI electrode localization in everyday clinical practice than the results here demonstrate.

Additional limiting factors address the extent to which the data can be compared. The bias reported are on parameters that were derived from Cartesian coordinates. Computation of a rigid transformation between automatic and manual electrode labels could specify and quantify the bias in more detail. However, this was not feasible as the data required was not available from the automatic tool. Another limitation is that solely 2D projections were used for the manual evaluation, while the automatic method processed 3D data. For a direct comparison, both methods should the spatial parameters from a 3D dataset. In this work the evaluation of 2D projections was chosen as a reference as it is the clinically established method.

### Effect of image quality

With the automatic IGCIP tool, it was possible to successfully analyze the electrode position in all 50 images. The image resolution parameters used here (voxel sizes 0.2 mm and 0.25 mm) and the image quality score had no influence on the results of the automatic evaluation compared to the manual evaluation. When evaluating the clinical usability of the IGCIP tool, it should be noted that only images absent of motion artifacts were analyzed in this study. In a preliminary examination of the tool, the presence of such artifacts resulted in an error message, and the frequency of DVT scans with motion artifacts was not low at 24% of the 123 subjects that were initially available. This would mean that in up to a quarter of the cases available for the evaluation of the electrode position would have to be conducted done manually. The presence of these motion artifacts would reduce the accuracy of the manual measurements in comparison to nominal scans. 

In this study, post-operative DVT scans were used instead of pre-operative scans to segment the intracochlear structures due an absence of pre-operative scans for a sizeable number of subjects. At our clinic, pre-operative scans are performed off-site at different radiology centers. These datasets were not completely been available at the time of the study, and available scans were performed with different acquisition parameters, which would have led to an additional bias in this study. The ability to segment the intracochlear structures with post-operative DVT scans demonstrated the versatility and usefulness of the IGCIP tool. Accuracy of the segmentation of the cochlear structures, including critical landmarks such as the mid-modiolar axis and the round window, may have been obscured by the metallic electrode artifacts in post-operative scans [27, 37]. From the use and analysis of the tool we believe that the IGCIP tool was found to be accurate in determining the CI electrode position even when the cochlear structures were segmented with post-operative scans [37].

### Selection of the mid-modiolar axis

It was demonstrated that the differences between the automatic and manual method in localization the electrode position were primarily due to a different selection of the mid-modiolar axis. The influence of the localization of this axis on both electrode modiolus distance and angular insertion depth is obvious, as it is a significant landmark for the measurement of these two parameters (see sections "Manual image analysis" and "Automatic image analysis"). Manual image evaluation in this work was performed in accordance with a consensus panel on a cochlear coordinate system [28], in which the mid-modiolar axis is perpendicular to a plane through the basal turn of the cochlea, with the helicotrema as the origin of this axis. If the mid-modiolar axis is not perpendicular to the basal plane, it is imprecise to choose the helicotrema as a reference point for determining EMD in the *basal* region of the cochlea. It is therefore more reasonable to define the EMD as the smallest distance of an electrode to the mid-modiolar axis, which is the z-axis of the modiolus shaped as a cylinder, as is done in the IGCIP tool. Thus, EMD measurements with the automated method appear to be more accurate than with the clinical manual method used here. Unfortunately, it was not possible to verify the selection of the mid-modiolar axis with the IGCIP software, because the tool did not plot the axis to the user. 

When determining the angular insertion depth aDOI, selecting the z-axis of the cylindrically shaped modiolus is less useful than for the EMD measurements. This is because in the case of an oblique mid-modiolar axis, the reference plane for measuring the aDOI would change depending on the cochlear turn. With the IGCIP tool, the angular depth of insertion is measured in the 0° plane that is the plane that contains both the mid-modiolar axis line and the center of the round window (correspondence with Jack Noble, Vanderbilt University). This reference plane contains the center of the mid-modiolar axis in the *basal* turn of the cochlear, in the case of an oblique mid-modiolar axis, this may explain the deviating aDOI data compared to the manual method, in which the *helicotrema* has been selected as the center of the axis [28].

To reduce the variability between different electrode localization techniques, methods such as the algorithm proposed Wimmer et al. [53] couldbe widely implemented to robustly detect critical landmarks as the mid-modiolar axis. There are also opportunities for future research to address the development of electrode location parameters that are independent of critical landmarks such as the mid-modiolar axis. For this purpose, an update of the consensus panel reflecting advances in automation capabilities would be appreciated.

## Conclusions

In all cases of DVT scans with a spatial resolution of 0.2 to 0.25 mm and absent motion artifacts, it was feasible to evaluate the electrode position with the IGCIP tool. Motion artifacts are not rare in our clinical practice (a quarter of cases), thus requiring a manual electrode localization when the post-operative DVT scan is used for segmentation of cochlear structures. There are systematic differences in the measurement of EMD and aDOI between the automatic and manual method, which is likely due to a different selection of the mid-modiolar axis. When controlling for the selection of the mid-modiolar axis the manual measurements and outputs of the tool are comparable. When measuring EMD, the IGCIP tool is superior to the manual method because the smallest possible distance to the axis is determined depending on the cochlear turn, whereas the manual method selects the helicotrema as the reference point rigidly. With respect to the measurement of aDOI, the IGCIP tool uses the center of the mid-modiolar axis in the *basal* plane of the cochlea (0° plane), which is not in accordance with the consensus panel on a cochlear coordinate system [28].

In conclusion, the IGCIP is a promising tool for the automated processing of CT and DVT images. The tool is able to detect key landmarks of the intracochlear structure and identify the location of the electrodes relative to these structures. Demonstrated by the methodology of this study, the functionality of being able to segment the cochlear with post-operative scans provides an additional benefit in circumstances where pre-operative scans are not available or are not of sufficient quality. The tool is equally as impacted as human rates in response to clinical anomalies such as motion artifacts in the images and systematic differences between measurements cause by the selection of the mid-modiolar axis. For general clinical use we recommend the addition of functionality to mitigate or deal with motion artifacts and a more streamlined approach for manual intervention of the selection of the mid-modiolar axis. Without usability with motion artifacts and measurements of aDOI according to the consensus approach, the IGCIP tool is not unrestricted ready for clinical use. For research purposes a translation of the outputs of the tool to the consensus paper on the cochlear coordinate system would be beneficial.

## Data Availability

The datasets generated and/or analyzed during the current study are not publicly available due to a confidential agreement with the sponsor of the study (Cochlear Ltd., Australia) but are available from the corresponding author on reasonable request.

## Abbreviations

CI: Cochlear Implant
CT: Computer Tomography
IGCIP: Image-guided Cochlear Implant Programming
EMD: Electrode-to-Modiolar axis Distance
aDOI: Angular Depth of Insertion
SD: Standard Deviation
DVT: Digital Volume Tomography
ST: Scala Tympani
SV: Scala Vestibuli
MOD: Mid-modiolar axis
DICOM: Digital Imaging and Communications in Medicine
E: Electrode
RW: Round Window
IQ: Image Quality
ICC: Intraclass Correlation Coefficient
CoI: Confidence Interval
LoA: Limits of Agreement
IR: Image Resolution
